# Brahma Is Required for Proper Expression of the Floral Repressor *FLC* in *Arabidopsis*

**DOI:** 10.1371/journal.pone.0017997

**Published:** 2011-03-21

**Authors:** Sara Farrona, Lidia Hurtado, Rosana March-Díaz, Robert J. Schmitz, Francisco J. Florencio, Franziska Turck, Richard M. Amasino, José C. Reyes

**Affiliations:** 1 Max Planck Institute for Plant Breeding, Cologne, Germany; 2 Centro Andaluz de Biología Molecular y Medicina Regenerativa (CABIMER), Consejo Superior de Investigaciones Científicas (CSIC), Seville, Spain; 3 Department of Biochemistry, University of Wisconsin, Madison, Wisconsin, United States of America; 4 Instituto de Bioquímica Vegetal y Fotosíntesis, Consejo Superior de Investigaciones Científicas (CSIC)-Universidad de Sevilla, Seville, Spain; Umeå Plant Science Centre, Sweden

## Abstract

**Background:**

BRAHMA (BRM) is a member of a family of ATPases of the SWI/SNF chromatin remodeling complexes from Arabidopsis. BRM has been previously shown to be crucial for vegetative and reproductive development.

**Methodology/Principal Findings:**

Here we carry out a detailed analysis of the flowering phenotype of *brm* mutant plants which reveals that, in addition to repressing the flowering promoting genes *CONSTANS* (*CO*), *FLOWERING LOCUS T* (*FT*) and *SUPPRESSOR OF OVEREXPRESSION OF CO1* (*SOC1*), BRM also represses expression of the general flowering repressor *FLOWERING LOCUS C* (*FLC*). Thus, in *brm* mutant plants *FLC* expression is elevated, and *FLC* chromatin exhibits increased levels of histone H3 lysine 4 tri-methylation and decreased levels of H3 lysine 27 tri-methylation, indicating that BRM imposes a repressive chromatin configuration at the *FLC* locus. However, *brm* mutants display a normal vernalization response, indicating that BRM is not involved in vernalization-mediated *FLC* repression. Analysis of double mutants suggests that BRM is partially redundant with the autonomous pathway. Analysis of genetic interactions between BRM and the histone H2A.Z deposition machinery demonstrates that *brm* mutations overcome a requirement of H2A.Z for *FLC* activation suggesting that in the absence of BRM, a constitutively open chromatin conformation renders H2A.Z dispensable.

**Conclusions/Significance:**

BRM is critical for phase transition in Arabidopsis. Thus, BRM represses expression of the flowering promoting genes *CO*, *FT* and *SOC1* and of the flowering repressor *FLC*. Our results indicate that BRM controls expression of *FLC* by creating a repressive chromatin configuration of the locus.

## Introduction

In eukaryotic cells DNA is wrapped around an octamer of histones to form the nucleosome fiber, the basic component of chromatin. DNA-histone complexes generate a barrier that reduces the accessibility of transcription factors and the general transcriptional machinery to DNA. Among the mechanisms that have evolved to overcome this barrier is chromatin remodeling. Chromatin remodelers, which have been referred to as chromatin remodeling machines (CRMs), are multi-subunit complexes that use the energy of ATP hydrolysis to modify DNA-histone interactions [1].

All ATP-dependent CRMs share the presence of a DNA-dependent ATPase of the SWI2/SNF2 family, which works as the enzymatic subunit of the complex. The proteins of this family have two conserved catalytic domains, a SNF2_N and a HelicC domain. Sequence analysis of these domains reveals their division into different subfamilies. In addition, other conserved domains often found in chromatin proteins, such as bromodomains, chromodomains, PHD domains, are also present within the same subfamily [1], [2], [3]. In Arabidopsis, there are 41 SWI2/SNF2-like proteins (e.g., Chromatin Database, www.chromdb.org [4]) divided into 18 subfamilies [2]. The SWI2/SNF2 subfamily is comprised of four proteins: BRAHMA (BRM) [5], SPLAYED (SYD) [6], CHR12 and CHR23 [2], [7]. In yeast and animals, the proteins of this subfamily are part of the SWI/SNF-type complexes [1], although no plant SWI/SNF complexes have yet been purified. Several lines of evidence suggest that BRM is the ATPase of at least one of the putative SWI/SNF complexes in Arabidopsis. First, BRM is the only protein from the SWI2/SNF2 subfamily that has a C-terminal bromodomain, which is also found in SWI2/SNF2 and Brahma proteins from yeast and Drosophila respectively. Second, the N-terminal region of BRM interacts with the Arabidopsis SWI3C and SWI3B proteins [5], [8]. These proteins are orthologues of the yeast SWI3 protein, another component of the SWI/SNF complex [9]. Third, both *brm* and *swi3c* mutants display very similar phenotypic characteristics [8], [10]. In addition, BRM is purified from Arabidopsis nuclei as part of a high molecular mass complex [5].

BRM has a crucial role in vegetative, embryonic and reproductive plant development [5], [8], [11], [12]. Expression profiling using 10-day-old *brm* and wild-type (WT) seedlings showed that only 1% of the genes were differentially expressed in *brm* [13]. However, when the same experiments were carried out with leaves from 14-day-old seedlings, the number of misregulated genes was more than 4% [14]. These different results could indicate tissue and stage specificity for BRM-mediated gene expression. BRM is also required for the floral transition. Four main genetic pathways have been described that control flowering in Arabidopsis: the photoperiod pathway (day lengths), the vernalization pathway (prolonged cold temperature experienced during winter), the gibberellin pathway (gibberellins) and the autonomous pathway (repression of *FLC*) [15], [16]. These different routes converge at the regulation of the integrator genes that play a crucial role in the regulation of floral transition. Transgenic plants with reduced expression of *BRM* (*BRM-*silenced plants) showed an early-flowering phenotype in long day and short day conditions (LD and SD respectively) and these results were correlated with an increase in the expression of the flowering integrator gene *FLOWERING LOCUS T* (*FT)* and the photoperiod-pathway gene *CONSTANS* (*CO)* [5]. *brm* mutants showed a most dramatic phenotype than *BRM-*silenced plants with a slow growth, delayed development and a strong plant size reduction. The *brm* mutants flowered with less leaves than WT plants, but a percentage of the mutant plants never flowered under SD [8]. These data indicate a more complex scenario for the involvement of BRM in flowering, which prompted us to carry out an in depth characterization. We show here that BRM is not only involved in regulation of the photoperiod pathway genes, but it is also an essential repressor of *FLOWERING LOCUS C* (*FLC)*.

## Materials and Methods

### Plant material and growth conditions

Wild-type *Arabidopsis thaliana*, T-DNA mutants and transgenic plants (all of them in Col-0 accession) were grown either in pots containing a mixture of substrate and vermiculite (3∶1) or aseptically in Petri dishes containing Murashige and Skoog media supplemented with 1% (wt/v) sucrose and 0.37% (wt/v) Phytagel (Sigma). Plants were grown in cabinets under long-day (16 h light/8 h dark) or under two different short-day conditions (10 h light/14 h dark or 8 h light/16 h dark). Short day experiments were performed under 10 h light/14 h dark except when indicated. Photoregimes at 22°C (day)/20°C (night), 70% relative humidity, and light intensity of 130 µE m^−2^s^−1^ were supplied by fluorescent lamps.

*brm-1*, *brm-2*, *ft-10*, *co-10*, *flc-3*, *fve-3*, *sef-2*, *pie1-5* mutants and *brm29-1*, *gCO::GUS* and *pFT::GUS* transgenic lines have been previously described [5], [8], [17], [18], [19], [20], [21], [22].

For vernalization treatments, seeds were germinated for 5 d at 22°C and vernalized for 40 d at 4°C under 8 h of light and 16 h of dark. Post-vernalization samples continued to grow plates under 8 h of light and 16 h of dark at 22°C.

### Gene expression analysis

RNA was isolated from whole seedlings using the RNeasy Mini Kit (Qiagen). 5 µg of RNA was used to generate first-strand cDNA with the SuperScript First-Strand Synthesis System for the RT-PCR kit (Invitrogen). Semi-quantitative PCR was performed using 2 µl of a 20 µl of RT reaction and a number of amplification cycles to be in the linear range of the reaction (15–25 cycles). DNA products were detected by Southern blot hybridization. For each experiment, three biological replicates were carried out and a representative one is shown. For quantitative RT-PCR (qRT-PCR), cDNA was diluted to 150 µl water and 3 µl diluted cDNA was used for subsequent reactions. Amplified products were detected using iQ™ SYBR® Green Supermix (Biorad) in an IQ5 (Biorad) thermal cycler. Data are mean of at least three biological replicates and three independent technical replicates were carried out for each data point. The primer pairs used for expression analyses are described in Table S1.

β-glucuronidase (GUS) activity was assayed as described in [5].

### Chromatin immunoprecipitation (ChIP) assays

ChIP assays were carried out using 1 g of 18-day-old seedlings grown in soil and crosslinked with 1% formaldehyde at room temperature for 15 min. After grinding the plant material with liquid nitrogen, chromatin was isolated as in [23] and sonicated to obtain an average fragment size of 0.2–1.2 kb. The chromatin solution was diluted 10-fold with ChIP dilution buffer (1.1% Triton X-100, 1.2 mM EDTA, 16.7 mM Tris-HCl pH 8, 167 mM NaCl) and precleared by incubating with protein-A agarose beads (SIGMA). To immunoprecipitate the histone-DNA complexes the following antibodies were used: anti-H3K4me3 (07-473; Millipore) and anti-H3K27me3 (07-449; Millipore). An equal amount of chromatin not treated with antibody was used as the mock antibody control and a small aliquot of untreated sonicated chromatin was used as the total input DNA control. Primers used for ChIP-PCR are described in Table S1.

### Statistics

When difference between the set of data were small, significance of the difference was estimated by determining the P value using a 2-sample Student's t-test (http://www.usablestats.com/calcs/2samplet).

## Results

### BRM represses the photoperiod pathway

**Figure 1:**
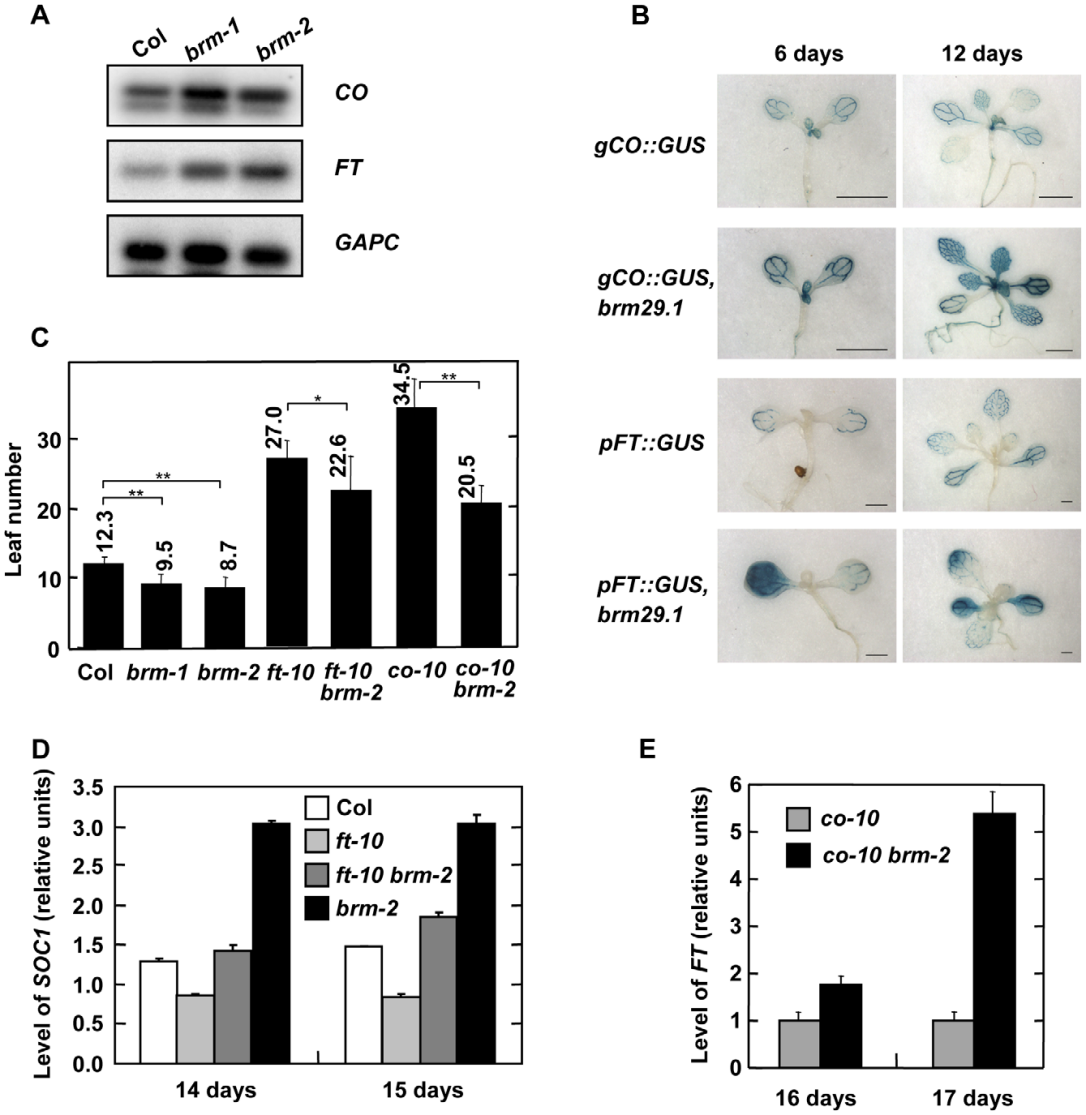
BRM controls expression of *CO*, *FT* and *SOC1* genes. A) Analysis of *CO* and *FT* expression in wild-type (Col), *brm-1* and *brm-2* mutant plants by RT-PCR. Total RNA was isolated from seedlings collected 10 h after dawn at 12 days of growth under LD conditions. *GAPC* transcript levels were also determined as a control for the amount of input cDNA. B) GUS expression patterns of *gCO::GUS* and *pFT::GUS* in wild-type and *BRM*-silenced plants (*brm29.1*) in whole-mount staining of 6-day-old and 12-day-old seedlings under LD conditions. C) Flowering time of plants grown under LD photoperiod. Data are means and standard deviation of at least 20 plants. Differences between the indicated pairs of data are significant with p<0.05 (*) or p<0.01 (**). D) Analysis of *SOC1* expression in wild type (Col), *ft-10*, *ft-10 brm-2* and *brm-2* plants by qRT-PCR. Total RNA was isolated from seedlings collected 10 h after dawn at 14 and 15 days of growth under LD conditions. E) Analysis of *FT* expression in *co-10* and *co-10 brm-2* mutant plants by qRT-PCR. Total RNA was isolated as in D.

We have previously shown that transgenic plants with reduced levels of BRM display higher levels of *CO* and *FT* transcripts compared to wild-type (WT) [5]. These results were confirmed in *brm-1* and *brm-2* mutant plants by RT-PCR experiments (Figure 1A). However, *TWIN SISTER OF FT* (*TSF*), the closest homolog of *FT* in Arabidopsis, was not up-regulated in *brm* mutants (data not shown). It has been demonstrated that both *CO* and *FT* are expressed in the vascular tissue of cotyledons and leaves [21]. In order to determine whether overexpression of *CO* and *FT* in the absence of BRM was restricted to the same tissue or whether, in contrast, both genes were ectopically overexpressed, we performed β-glucoronidase (GUS) staining of plants expressing *pFT::GUS* and *gCO::GUS* [21] in *BRM*-silenced plants (*brm29.1*). Figure 1B shows that GUS activities of both reporter constructs were significantly increased in *BRM*-silenced plants. However, while *gCO::GUS* expression was restricted to the vascular tissue, both in WT and *BRM-*silenced plants, *pFT::GUS* was ectopically expressed in the plants with reduced levels of BRM. These results suggest that BRM is affecting transcriptional repression of both *CO* and *FT*, which is consistent with the early-flowering phenotype of the *BRM*-silenced and the *brm* mutant plants [5], [8].

Next, we examined whether absence of FT or CO could suppress the early-flowering phenotype of *brm* plants. Similarly to *ft-10* plants, the *ft-10 brm-2* double mutants resulted in a late-flowering phenotype, although the *ft-10 brm-2* plants flowered slightly earlier (Figure 1C). These data suggest that *BRM* is mainly but not only upstream of *FT* in the floral promotion pathway. The slight early phenotype could be due to *SOC1* expression. To test this hypothesis we measure the level of *SOC1* mRNA in *ft-10*, *brm-2* and *ft-10 brm-2* plants by qRT-PCR. Thus, whereas *SOC1* was strongly up-regulated in the *brm-2* mutant, its expression was reduced in *ft-10 brm-2* plants, but still higher than in *ft-10* plants (Figure 1D). Interestingly, *co brm-2* plants flowered later than *brm-2* mutants but significantly earlier that *co* mutants, suggesting that BRM is controlling *FT* through *CO* repression but also independently of *CO*. To verify this point, we determined the levels of *FT* mRNA in the *co* and the *co brm-2* mutants. Levels of the *FT* transcript were increased in *co brm-2* plants compared to the levels observed in *co* plants (Figure 1E). Taken together, these results confirm that BRM can control *FT* independently of CO and *SOC1* independently of FT and, therefore, is able to act at different levels of the photoperiod pathway.

### BRM represses *FT* independently of FLC

**Figure 2:**
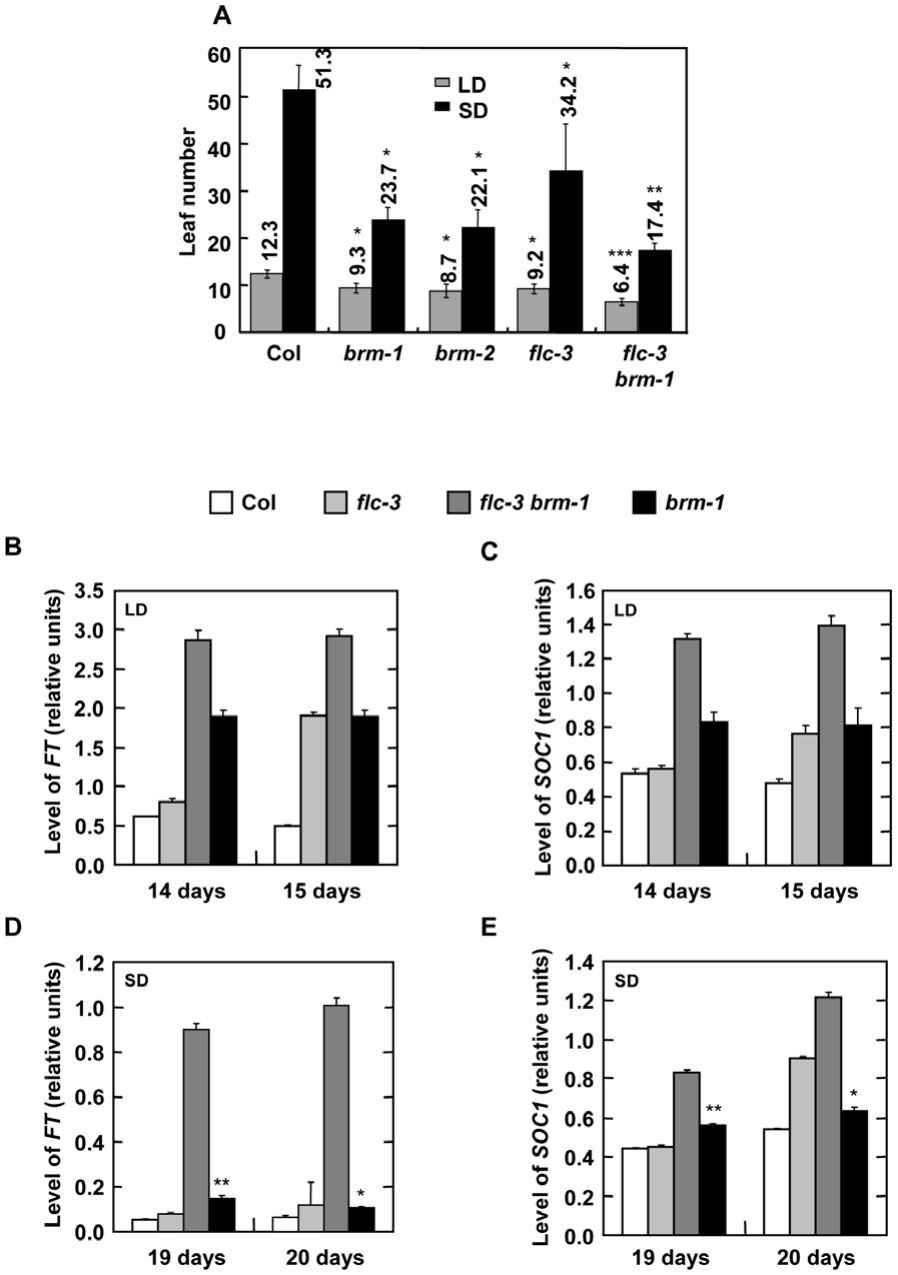
*flc-3* mutation enhances the early flowering phenotype of *brm* mutants. A) Flowering time of plants grown under LD or SD photoperiod. Data are means and standard deviation of at least 20 plants. Asterisks indicate significant differences between Col and *brm-1*, *brm-2* and *flc-3* with p<0.01 (*) for both LD and SD data, or between *flc-3 brm-1* and the other background with p<0.001 (**) for SD and p<0.00001 (***) for LD data. B) Analysis of *FT* expression in wild-type (Col), *flc-3*, *flc-3 brm-1* and *brm-1* plants by qRT-PCR. Total RNA was isolated from seedlings collected 10 h after dawn at 14 and 15 days of growth under LD conditions. C) Analysis of *SOC1* expression as in C. D) Analysis of *FT* expression in wild-type (Col), *flc-3*, *flc-3 brm-1* and *brm-1* plants by qRT-PCR. Total RNA was isolated from seedlings collected 10 h after dawn at 19 and 20 days of growth under SD conditions. E) Analysis of *SOC1* expression as in D. Asterisks indicate significant differences between wt and *brm-1* with p<0.005 (*) or p<0.02 (**).

FLC binds to the *FT* gene, which results in direct repression of expression of the gene [24], [25]. Our results indicate that BRM is also a repressor of *FT*. One possibility is that BRM cooperates with FLC in the repression of *FT*. To investigate this possibility we constructed *flc-3 brm-1* double mutants and analyzed their flowering-time phenotype. Under long day conditions, *flc-3* plants flowered with about 9 leaves, similar to the number of leaves that the *brm-1* and *brm-2* plants displayed (Figure 2A). Interestingly, *flc-3 brm-1* plants flowered earlier than the single mutants (6.4±0.8 leaves). The enhanced early flowering was even more extreme in short days (23.7±2.8 and 34.2±10.1 leaves in *brm-1* and *flc-3* plants, respectively, versus 17.4±1.45 leaves in *brm-1 flc-3*). Since both *flc-3* and *brm-1* are null alleles, this additive phenotype suggested that BRM represses *FT* independently of FLC. This hypothesis was confirmed by expression analysis. *FT* is up-regulated in *brm-1* and *flc-3* mutants, but this up-regulation is stronger in the *flc-3 brm-1* plants and the same was observed for *SOC1* (Figure 2B–2C). Therefore, BRM acts upon *FT* and *SOC1* through an FLC-independent pathway.

### Expression of *FLC* is increased in *brm* plants

**Figure 3:**
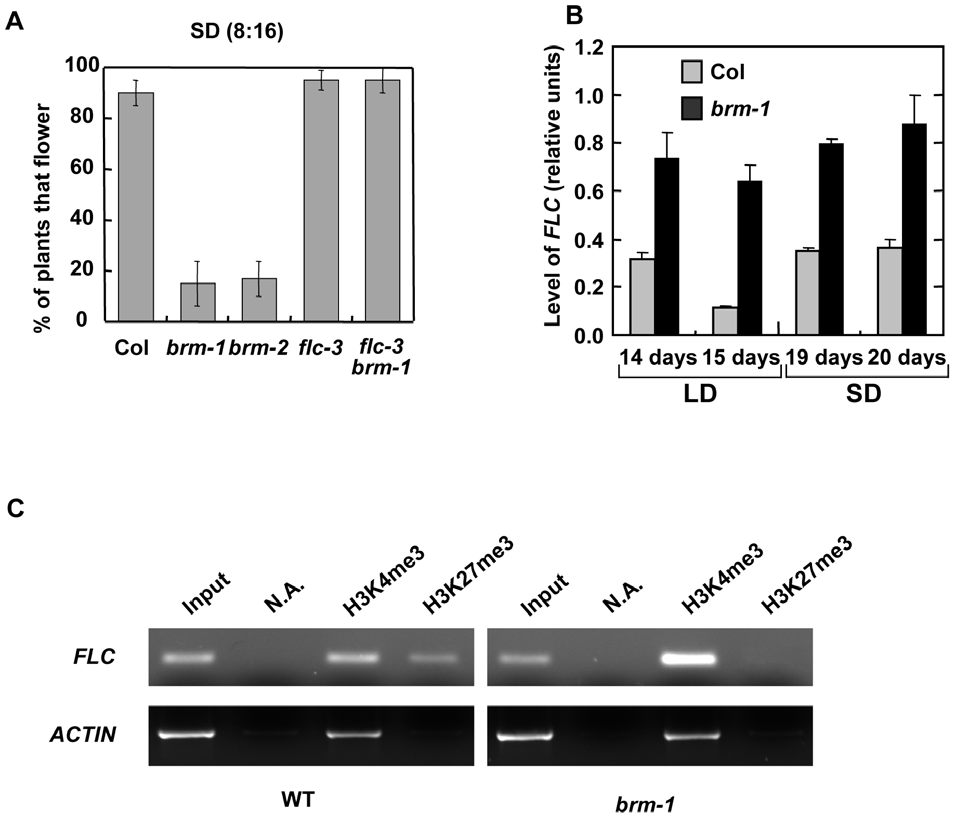
BRM controls expression of *FLC*. A) Percentage of flowering under SD (8∶16) conditions of wild-type (Col), *brm-1*, *brm-2*, *flc-3* and *brm-1flc-3* plants. B) Analysis of *FLC* expression in wild-type (Col) and *brm-1* plants by qRT-PCR. Total RNA was isolated from seedlings collected 10 h after dawn at 14 and 15 days of growth under LD conditions and at 19 and 20 days under SD conditions. C) Analysis of the levels of H3K4me3 and H3K27me3 by ChIP-PCR at the *FLC* promoter in WT and *brm-1* mutant plants. A representative experiment of three independent replicates is shown.

We have previously reported that about 20% of *brm-1* and *brm-2* plants never flower in short days (10 hours light/14 hours dark) [8]. To further investigate this phenomenon we decided to cultivate the plants under a more restrictive short day condition (8 hours light/16 hours dark). Under this light regimen only about 15% of the *brm* mutant plants flowered after 90 days of culture (Figure 3A). These data suggested that in the absence of signalling from the photoperiod-dependent pathway some factors were repressing flowering in the absence of BRM. An obvious candidate to test was the floral repressor FLC based on our observations reported above. Interestingly, the *flc-3* mutation was able to suppress the non-flowering phenotype of the *brm-1* mutant plants (Figure 3A). Furthermore, the levels of *FLC* mRNA were significantly increased in *brm-1* both in LD and SD (Figure 3B). In contrast, transcript levels of the gene next to *FLC, UPSTREAM OF FLC (UFC)* were not affected by the absence of BRM (Figure S1). These data indicate that BRM is a repressor of *FLC*. To fully understand why *brm* mutants do not flower under SD, *FT* and *SOC1* expression was also analyzed under these conditions. Interestingly, despite of the increased expression of *FLC* and the lack of signalling from the photoperiod pathway in SD, levels of *FT* and *SOC1* were slightly, but significantly increased in the *brm-1* plants under these conditions (Figure 2D and 2E). Therefore, *FT* and *SOC1* repression by FLC overexpression in *brm* plants is not sufficient to explain the no-flowering phenotype of the mutant in SD. However, the strong upregulation of *FT* and *SOC1* in the double *flc-3 brm-1* mutant could be the reason of the suppression of the no-flowering phenotype in these plants.

Since BRM may work by altering the chromatin configuration of the genes that represses, we decided to analyze how the absence of BRM affects posttranscriptional histone modifications such as the active mark H3K4me3 and the repressive mark H3K27me3 in *FT* and *FLC* loci. Whereas in *FT* locus there were not significant changes (data not shown), chromatin immunoprecipitation experiments demonstrated that the promoter region of *FLC* displays increased levels of H3K4me3 in the *brm-1* plants compared to WT (Figure 3C). Increased levels of H3K4me3 have been found in other genotypes with increased levels of *FLC* expression such as Col *FRI*, *fld* and *fve* [26], [27]. H3K27me3 is a repressive mark introduced by a multiprotein complex functionally and structurally related to the animal Polycomb Repressor Complex-2 (PRC2) [28]. Recent studies have shown that the Arabidopsis PRC2 subunits, including CLF, FIE and EMF2, repress *FLC* expression in plants grown under normal conditions (without vernalization treatment) by promoting H3K27 methylation of the *FLC* chromatin [26]. Interestingly, levels of H3K27me3 at the *FLC* promoter were reduced in *brm* plants with respect to WT plants (Figure 3C), suggesting that BRM can cooperate directly or indirectly with the Polycomb complex in repressing *FLC* in non-vernalized conditions.

### BRM is not required for the vernalization response

**Figure 4:**
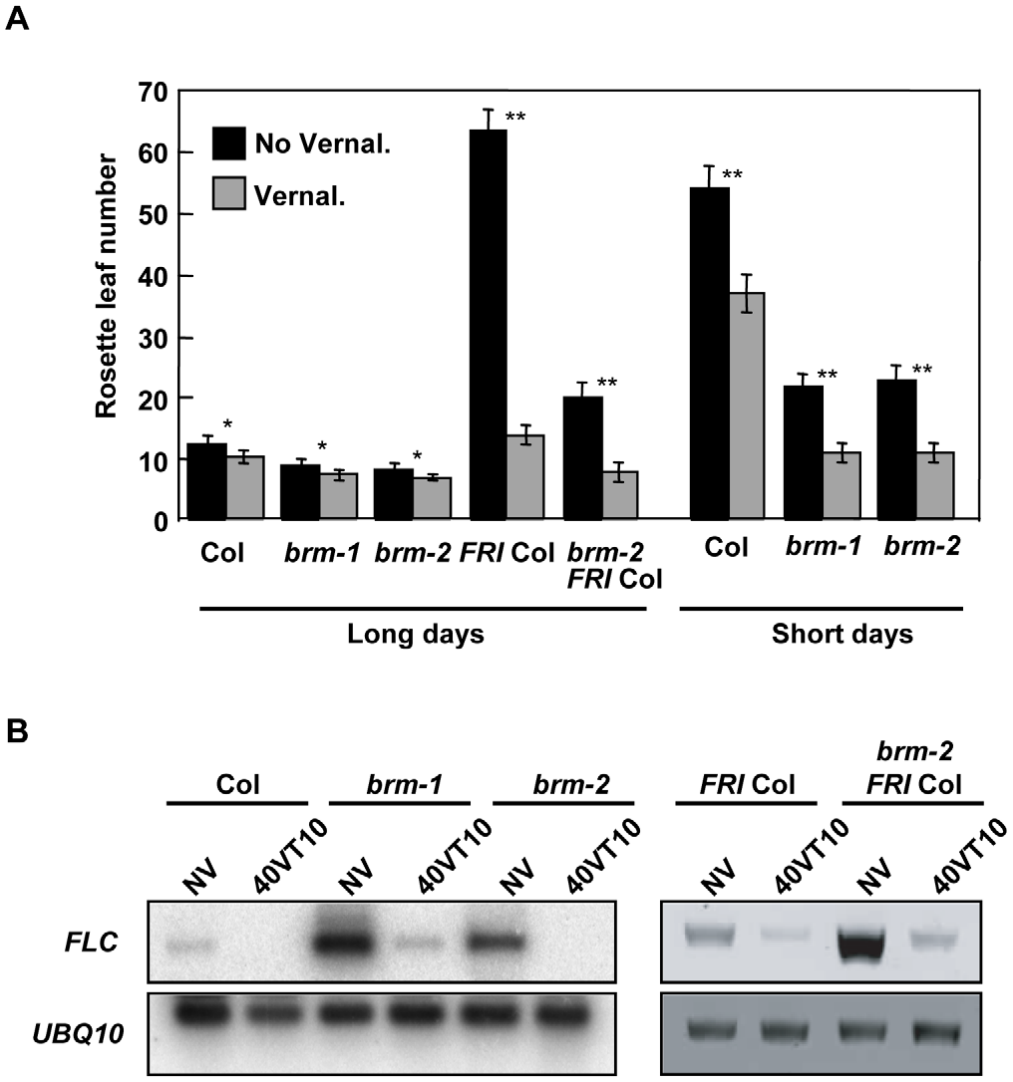
BRM is not required for the vernalization response. A) Flowering behaviour of *brm* mutants with and without vernalization in long days and short days. Plants were vernalized for 40 days, then transferred either to LD or to SD (as indicated) conditions and flowering time was determined. Data are means and standard deviation of at least 15 plants. Differences between vernalized and not vernalized set of data were significant with p<0.05 (*) or p<0.00001 (**). B) Analysis of *FLC* expression by RT-PCR of vernalized or not vernalized plants. Total RNA was isolated from non-vernalized seedlings (NV) or 10 days after transferring vernalized plants to LD normal conditions (40VT10).

The vernalization pathway is required to maintain low levels of FLC after a prolonged cold treatment (recently reviewed in [22]). This repression is epigenetically maintained during the subsequent development of the plant. Silencing of *FLC* during vernalization is mediated by a vernalization-specific PRC2 complex. Our experiments suggested that BRM was a repressor of *FLC* expression. Therefore, we decided to investigate whether BRM was required for *FLC* silencing during vernalization. The *brm-2* allele was crossed into a line containing an active *FRIGIDA* allele. WT and *brm* mutants both in the Col and the Col;*FRI* background were vernalized for 40 days then transferred either to long days or to short days (only Col plants) and flowering time was measured (Figure 4A). Interpretation of the flowering data was complicated by the fact that levels of *FT* transcripts are increased in the *brm* background and therefore *brm* plants flower earlier than WT plants irrespective of vernalization. Nevertheless, acceleration of flowering by vernalization was clearly observed both in the presence and in the absence of BRM in the Col background and in the Col;*FRI* background. Consistently, levels of the *FLC* transcript were normally reduced by cold, both in the presence and in the absence of BRM (Figure 4B). These data indicate that BRM is not required for vernalization-induced silencing of *FLC*.

### BRM and the autonomous pathway

**Figure 5:**
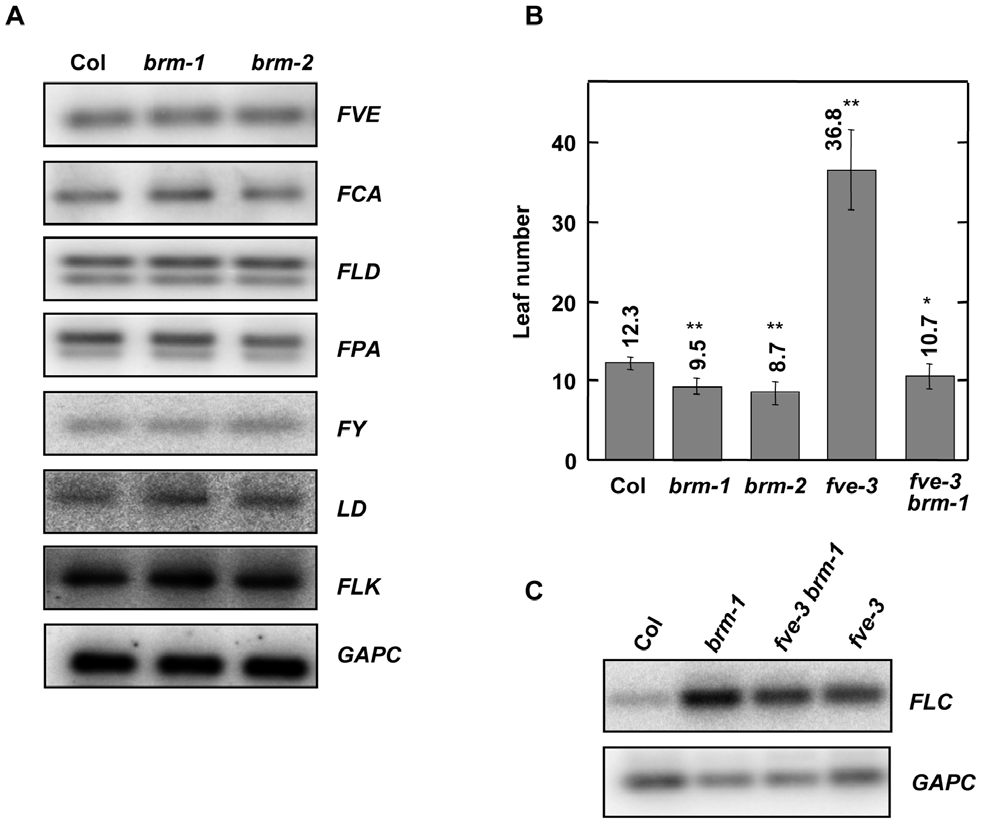
Interaction of BRM with the autonomous pathway. A) Analysis of *FVE, FCA, FLD, FPA, FY, LD*, and *FLK* expression in wild-type, *brm-1* and *brm-2* mutant plants by RT-PCR. Total RNA was isolated from seedlings collected 10 h after dawn at 12 days of growth under LD conditions. *GAPC* transcript levels were also determined as a control for the amount of input cDNA. B) Flowering time of plants grown under LD photoperiod. Data are means and standard deviation of at least 20 plants. Asterisks indicate significant differences between Col and the mutant backgrounds with p<0.00002 (*) or p<0.00001 (**). C) Analysis of *FLC* expression in wild-type, *brm-1*, *fve-3* and *fve-3 brm-1* mutant plants by RT-PCR. Total RNA was isolated as indicated in A. *GAPC* transcript levels were also determined as a control for the amount of input cDNA.

The autonomous pathway was originally defined by late-flowering mutants that retain a photoperiod and a vernalization response [29]. Later on it became clear that all autonomous-pathway members are repressors of *FLC* [20]. *brm* mutants are not late flowering in LD due to the upregulation of *FT*; however, expression of *FLC* is repressed by BRM and *brm* mutant plants respond normally to vernalization and thus, *BRM* could be considered an autonomous-pathway component. The classic components of the autonomous pathway include FCA [30], FPA [31], FLK [32], [33], FVE [17], [34], FLD [35], LD [22], FY [36]. One possibility to explain the effect of BRM mutations on *FLC* is that BRM activates the expression of an autonomous-pathway component. We evaluated this possibility by comparing the mRNA levels of *FVE*, *FLD*, *FCA*, *FPA*, *LD*, *FY*, and *FLK* genes between wild-type and *brm* mutant plants. As shown in Figure 5A the mRNA levels of all of the classic autonomous-pathway genes were not significantly affected in *brm* plants. FVE, a homolog of the human mammalian RbAp46/48 which has been found in several chromatin-modifying repressor complexes, is involved in histone deacetylation of the *FLC* chromatin [17]. Since BRM may work by altering chromatin configuration of the *FLC* locus we decided to investigate the genetic interaction between *BRM* and *FVE*. To do that a *brm-1 fve-3* double mutant was constructed. As expected, *fve-3* plants presented a late-flowering phenotype and displayed up-regulation of *FLC* [17], [34]. This late-flowering phenotype was suppressed by the *brm* mutation due to the fact that BRM controls *FT* downstream of *FLC* (Figure 5B). Therefore, in order to investigate the interaction between BRM and FVE on the regulation of *FLC*, we determined the levels of the *FLC* mRNA by RT-PCR. Levels of *FLC* mRNA were not increased in the double *brm-1 fve-3* mutant compared to the single mutants indicating that there were not additive interactions (Figure 5C), and therefore suggesting that BRM cooperates at least with one autonomous-pathway component (*FVE*) in controlling *FLC* expression.

### The SWR1 complex is not required for expression of *FLC* in the absence of BRM

**Figure 6:**
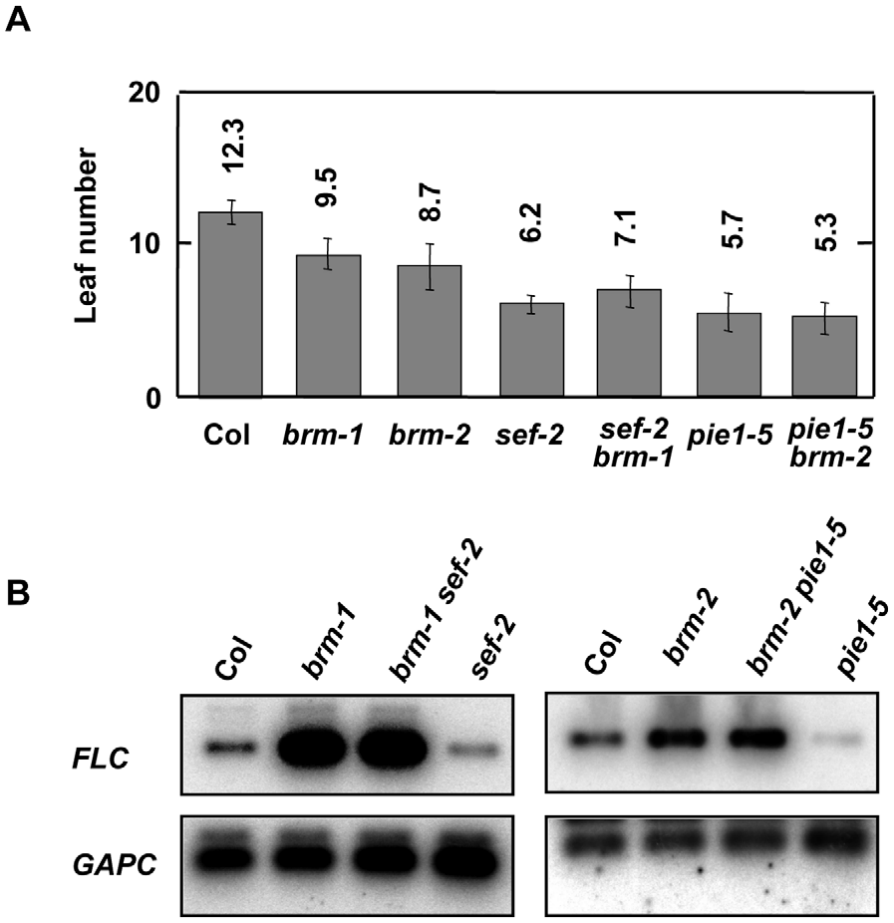
SEF and PIE1 are not required for expression of *FLC* in the absence of BRM. A) Flowering time of plants grown under LD photoperiod. Data are means and standard deviations of at least 20 plants. B) Analysis of *FLC* expression in wild-type, *brm-1*, *brm-2 sef-2*, *pie1-5*, *sef-2 brm-1* and *pie1-5 brm-1* mutants by RT-PCR. Total RNA was isolated from seedlings collected 10 h after dawn at 12 days of growth under LD conditions. *GAPC* transcript levels were also determined as a control for the amount of input cDNA. Col data were significantly different to *brm-1* and *brm-2* data with p<0.01. Col data were significantly different to *sef-2*, *sef-2 brm-1*, *pie1-5*, *pie1-5 brm-2* data with p<0.001. *brm-1* and *brm-2* data were different to *sef-2, sef-2 brm-1, pie1-5, pie1-5 brm-2* data with p<0.01.

Several groups, including ours, have demonstrated that the Arabidopsis SWR1 complex is required for expression of *FLC* (recently reviewed in [37]). Therefore, mutations in genes encoding components of the complex lead to reduced levels of *FLC* expression, which result in an early flowering phenotype [19], [27], [38], [39], [40], [41], [42], [43]. Furthermore, SWR1 subunit mutants such as *pie1-1* and *eds1/arp6* are able to suppress the late-flowering phenotype of Col;*FRI* and autonomous-pathway mutants [27], [43]. This suppression was accompanied by a reduction in the levels of *FLC* mRNA indicating that the SWR1 complex is required for the increased *FLC* expression that results from the presence of *FRI* or from mutations in components of the autonomous pathway. Therefore, we decided to investigate whether mutations in the SWR1 components are able to suppress the *FLC* up-regulation that occurs due to loss of BRM. We generated *brm-2 pie1-5* and *brm-1 serrate leaves and early flowering-2 (sef-2)* double mutants. Double *brm-2 pie1-5* and *brm-1 sef-2* plants flowered slightly but significantly earlier than single *brm* mutants and about the same time as *pie1-5* and *sef-2* single mutant plants (Figure 6A). More importantly, an increase in the amount of *FLC* transcription observed in *brm* mutants was not suppressed either by *sef-2* or by *pie1-5* (Figure 6B). These data suggest that the absence of BRM results in a chromatin configuration at the *FLC* locus that bypasses the requirement of the SWR1 complex for the expression of *FLC*.

## Discussion

The different genetic pathways that control flowering are well defined and it is known that chromatin structure plays an important role in such regulation [44]. BRM is an ATPase of the SWI2/SNF2 family and a possible component of a plant SWI/SNF chromatin remodeling complex. In animals, the different components of these complexes play an essential role in development and their mutations result in altered developmental patterns, cancer and embryo lethality. *brm* mutants are not lethal, although they are sterile due to gametophytic defects and they have pleiotropic phenotypes affecting the embryo as well as the adult plant [5], [8], [10], [12], [14]. Among these phenotypes, *brm* mutants and also *BRM*-silenced lines have an altered flowering behaviour [5], [8]. Here we have further elucidated the flowering pathways affected by loss of BRM.

### BRM is a repressor of the photoperiod pathway and the floral integrator genes *FT* and *SOC1*

**Figure 7:**
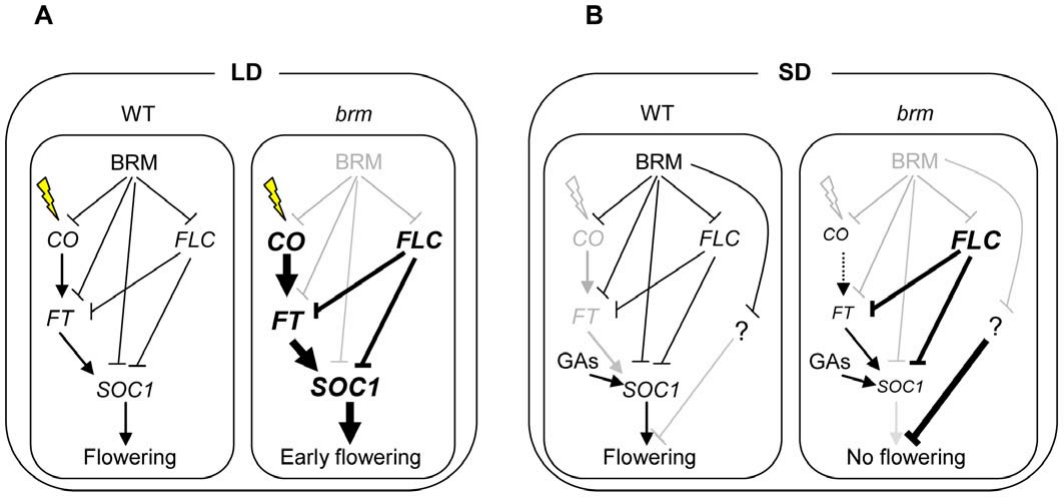
Model for the role of BRM in flowering regulation. A) Under LD conditions in WT plants the photoperiod pathway overcomes the repression mediated by BRM upon *CO*, *FT* and *SOC1* to promote flowering. In a *brm* mutant, the high levels of expression of *CO*, *FT* and *SOC1* leads to an early flowering phenotype in spite of the increase in *FLC* expression. B) Under SD conditions in WT plants, the photoperiod pathway is not induced and flowering relies on the activation of *SOC1* by the GAs pathway; however in *brm* plants, although *FT* and *SOC1* are still slightly up-regulated in spite of the strong *FLC* expression, flowering is not induced indicating that there are other pathways (“?” in the scheme) that are also repressed by BRM.

*CONSTANS* (*CO*) is a key component in the promotion of flowering by long days. CO main function is the activation of *FT* in the leaves. FT moves from the leaves to the apical meristem to trigger a cascade of events that will lead to the flowering of the plant. One of the earliest events is the activation of *SOC1* expression [16], [45]. Here we show that the three genes, *CO*, *FT* and *SOC1* are up-regulated in *brm* mutant lines (Figure 1) raising the question of whether BRM controls these genes dependently or independently of each other. Our genetic data show that the early-flowering phenotype of *brm* mutants is almost, but not completely reverted in a *ft* background (Figure 1C), suggesting that *FT* mostly contributes to the early-flowering phenotype of *brm*. However, in the *ft-10 brm-2* double mutant *SOC1* is slightly up-regulated, indicating that *SOC1* is also involved in this phenotype and that BRM is able to repress *SOC1* independently of *FT*. Besides, in a *co* mutant background the *brm* early-flowering phenotype is partially rescued. Therefore, the regulation of *FT* by BRM also takes place in at least two different ways: through *CO* repression and independent of *CO* (Figure 7). In summary, our results highlight the complexity of the interactions between BRM and the different components of the photoperiod pathway.

*FT* expression is tightly regulated in the leaves where it is only expressed in the companion cells of the phloem of the apical part of cotyledons and leaves [21]. *CO* is also expressed in the veins of cotyledons and leaves, but more broadly than *FT* (Figure 1B; [21], [46]. In *BRM*-silenced plants, *CO* expression is still limited to the veins, although a clear up-regulation is observed. On the other hand, *FT* is ectopically expressed, but the overexpression is not as strong and general as in a *35S::CO* background (Figure 1B; [21]), indicating that BRM repression is necessary for the tissue specificity of *FT* expression.

### BRM is an essential player in *FLC* repression

*FLC*, which encodes the main repressor of flowering in Arabidopsis has become a model gene in the study of chromatin regulation [28], [44], [47]. Despite the flowering phenotype of *brm* mutants, a percentage of the mutant plants never flowered under non-inductive conditions what indicated that other players were also involved in *brm* flowering phenotype. Indeed, *FLC* is up-regulated in *brm* mutants in LD and SD conditions (Figure 3B). However, level of *FT* and *SOC1* are slightly, but still significantly up-regulated in *brm* plants under SD compared with WT plants, suggesting that a strong repression of these genes due to the increased levels of FLC is not the cause of the no-flowering phenotype of the *brm* plants. In SD *brm* mutants show a more dramatic phenotype than in LD (Figure S2) and, therefore, other developmental key pathways might be affected preventing the flowering transition (Figure 7). The no-flowering phenotype of *brm* plants in SD is completely suppressed in the double *flc-3 brm-1* mutant probably due to the up-regulation of *FT* and *SOC1*. Despite the fact that our genetic data demonstrate that FLC and BRM act independently on *FT* expression we see a synergic activation of *FT* under SD in the double *flc-3 brm-1* mutant, suggesting that FLC and BRM may display overlapping repressing roles. In addition, other developmental phenotypes are also suppressed in the absence of FLC, indicating that the up-regulation of *FLC* plays a main role in the phenotypes observed in *brm* grown under SD.

When *brm* plants are grown in long days the strong up-regulation of *FT* induces early flowering despite *FLC* up-regulation. However, in the absence of *FT*, *brm* does not flower later as it should be expected due to the increased *FLC* expression. The most probable reason is the slight up-regulation of *SOC1* in *ft-10 brm-2* plants (Figure 1D). Therefore, the absence of BRM is able to overcome the lack of FT and a higher amount of FLC, activator and repressor of *SOC1* respectively.

Activation of *FLC* is mediated by FRI and two different hypotheses have been proposed recently to explain its molecular function; the first one involves FRI-mediated histone methylation of *FLC* chromatin and the second one proposes FRI is important for *FLC* RNA processing [48], [49]. Absence of BRM increases the levels of *FLC* expression even in the absence of wild-type FRI (Columbia background). Furthermore, the absence of BRM and presence of a WT FRI allele have an additive effect on *FLC* expression levels (Figure 4B), indicating that BRM and FRI act independently. *FLC* expression also requires the activity of the SWR1 complex, involved in the deposition of the H2A.Z histone variant, that has been involved in the perception of temperature [50]. PIE1, the catalytic subunit of this complex, is also a DNA-dependent ATPase of the SWI2/SNF2 family [43]. The SWR1 complex is needed for *FLC* expression even in accessions with an active FRI [37]. Our genetic data show that although mutations in the SWR1 complex components *PIE1* and *SEF* are epistatic on *brm* mutants, the effects on *FLC* expression of an impaired SWR1 complex are overcome by *brm* mutations (Figure 6). Considering that the SWR1 complex also regulates the expression of the flowering repressor genes *MAF4* and *MAF5* [51], [52], [53], the flowering data could be independent of *FLC*. Strikingly, the expression data suggest that in the absence of BRM, H2A.Z is not required for the expression of *FLC*. This is consistent with the proposed role for H2A.Z in transcription by poising genes for activation [54]. Thus, BRM would establish a repressive chromatin conformation where inclusion of H2A.Z would be essential for activation, but in the absence of BRM, the constitutively open chromatin conformation makes H2A.Z superfluous. Considering this hypothesis, the role of H2A.Z as a sensor of temperature fluctuations [50] and that such fluctuations overcome FLC-mediated flowering repression [55], in the future it will be very interesting to analyze if the absence of BRM will remove the plasticity in the response to different temperatures.

*FLC* is repressed by two main pathways, the vernalization and the autonomous pathways. In vernalized plants, *FLC* is repressed in response to exposure to prolonged low temperatures and such repression is stably maintained after the cold treatment by the Polycomb VRN2-complex what involves an increase in the levels of H3K27me3 at this gene [28], [44], [48]. A second mechanism, that seems to be mediated by another PRC2, the EMF2-complex, is also responsible of the deposition of this repressive histone mark in *FLC* independently of vernalization [26], [56], [57], [58], [59]. Mutations in *BRM* do not affect the vernalization-mediated repression of *FLC*, discarding a possible role of this protein in such regulation, but a reduction in the amount of H3K27me3 in non-vernalized plants was observed (Figure 3 and 4). Although this could be an indirect effect of the up-regulation of *FLC*, we cannot discard a more direct role of BRM in the deposition of this mark at *FLC* chromatin independently of vernalization and, therefore, more related with the EMF2-complex pathway.

The autonomous pathway is comprised of several components divided in different groups some of them related with RNA processing and others with chromatin regulation, but all of them share a role in *FLC* repression. BRM is not a regulator of this pathway, because the expression of autonomous pathway components is not affected in *brm*. Nevertheless, our results uncover a clear functional relationship between BRM and the autonomous pathway in the repression of *FLC*. For example, the analysis of the double *fve-3 brm-1* mutant showed functional redundancy between BRM and the autonomous-pathway component FVE (Figure 5). In addition, in *brm* mutants there is an increase in the amount of H3K4me3 at the *FLC* locus, as was previously shown for the *fve* and *fld* mutants [27], [60]. Furthermore, a physical interaction between AtSWI3B, a SWI/SNF subunit that interacts with BRM [8], and FCA, another autonomous pathway component, has been also demonstrated [10]. However, in contrast to autonomous-pathway mutants, *brm* mutations suppress the low levels of expression of *FLC* of SWR1 complex mutants. In this scenario, it is tempting to speculate that a BRM-containing complex could be required as the CRM necessary to set the right chromatin conformation that would allow changes of epigenetic modifications, linking the autonomous pathway with ATP-dependent chromatin remodeling.

In past few years many publications have shown the complexity of flowering regulation through chromatin factors that are involved in activation as well as in repression. Therefore, phenotypic analysis of mutants alone is not sufficient to clarify their specific role in the floral transition. This is due to the broad action of proteins involved in chromatin and epigenetic regulation of gene expression. For instance, TFL2/LHP1 and CLF are two known examples that function as repressors and activators of flowering by acting as repressor of both *FT* and *FLC* [26], [57], [58], [59]. Similarly, BRM is controlling flowering by repressing *FT* and *FLC*. We have seen a strong decrease in the levels of H3K27me3 at the *FLC* locus in the absence of BRM. Both CLF and TFL2/LHP1 are involved in the establishment and the maintenance of this epigenetic mark and in the repression associated to it. Further experiments are required to elucidate whether BRM, CLF and TFL2/LHP1 are in the same gene repression pathway in Arabidopsis.

## Supporting Information

Figure S1 ***UFC*** **is not up-regulated in** ***brm*** **mutants.** Analysis of *FLC* and *UFC* expression in wild-type, *brm-1* and *brm-2* mutant plants by RT-PCR. Total RNA was isolated from seedlings collected 10 h after dawn at 12 days of growth under LD conditions. *GAPC* transcript levels were also determined as a control for the amount of input cDNA.
(TIF)

Figure S2 ***FLC*** **is important for** ***brm*** **phenotypes in SD.** A) *flc-3*, *flc-3 brm-1* and *brm-1* plants grown under SD conditions. B) and C) Closer pictures of *brm*-*1* plants showing the dramatic characteristic phenotypes of the mutant grown under SD photoperiod.
(TIF)

Table S1 **List of primers.**
(TIF)
