# Supplementary figures and images for: Brahma Is Required for Proper Expression of the Floral Repressor FLC in Arabidopsis
Source: PLoS One. 2011 Mar 21;6(3):e17997. doi: 10.1371/journal.pone.0017997 (PMC3061888; doi:10.1371/journal.pone.0017997)

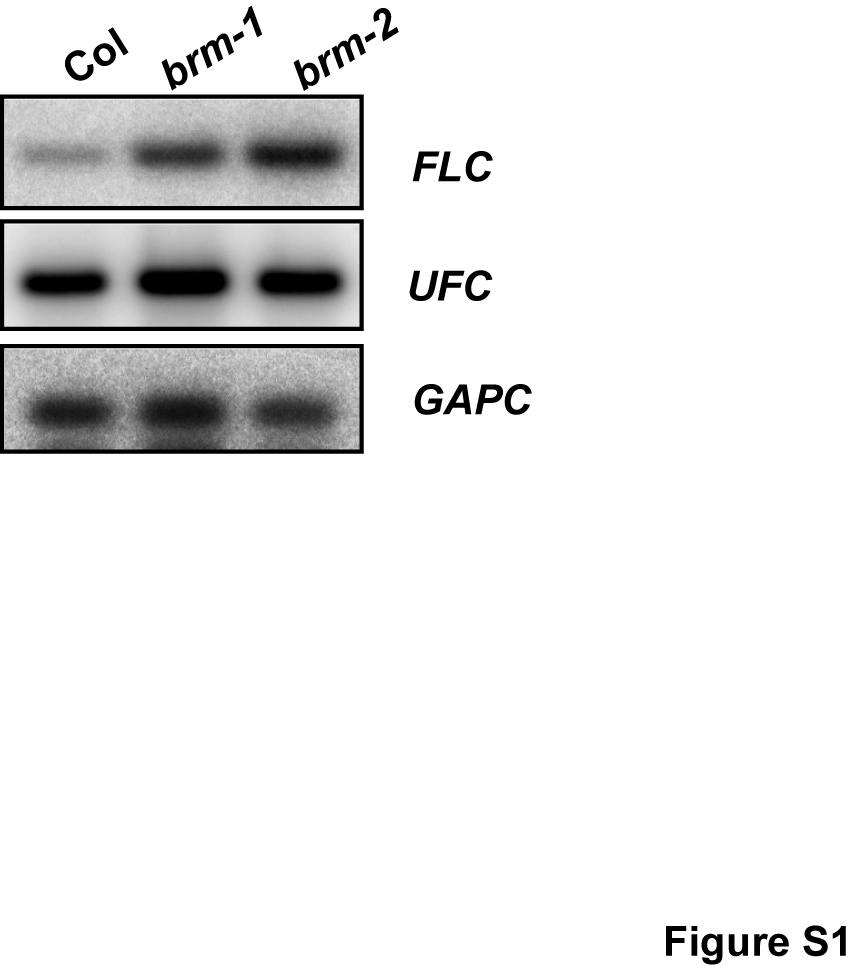

Supplement: Figure S1 — UFC is not up-regulated in brm mutants. Analysis of FLC and UFC expression in wild-type, brm-1 and brm-2 mutant plants by RT-PCR. Total RNA was isolated from seedlings collected 10 h after dawn at 12 days of growth under LD conditions. GAPC transcript levels were also determined as a control for the amount of input cDNA. (TIF) [file pone.0017997.s001.tif]

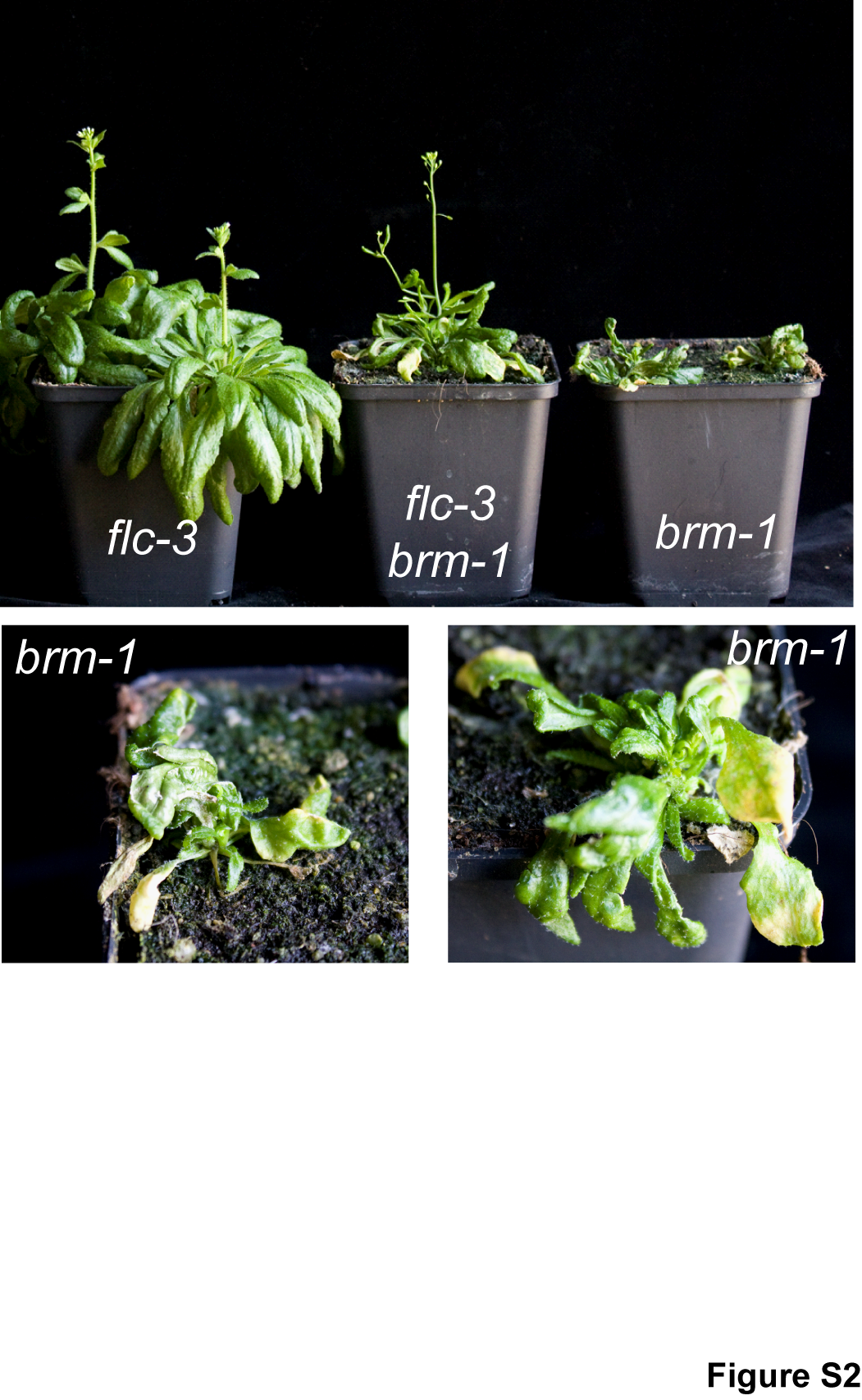

Supplement: Figure S2 — FLC is important for brm phenotypes in SD. A) flc-3, flc-3 brm-1 and brm-1 plants grown under SD conditions. B) and C) Closer pictures of brm-1 plants showing the dramatic characteristic phenotypes of the mutant grown under SD photoperiod. (TIF) [file pone.0017997.s002.tif]

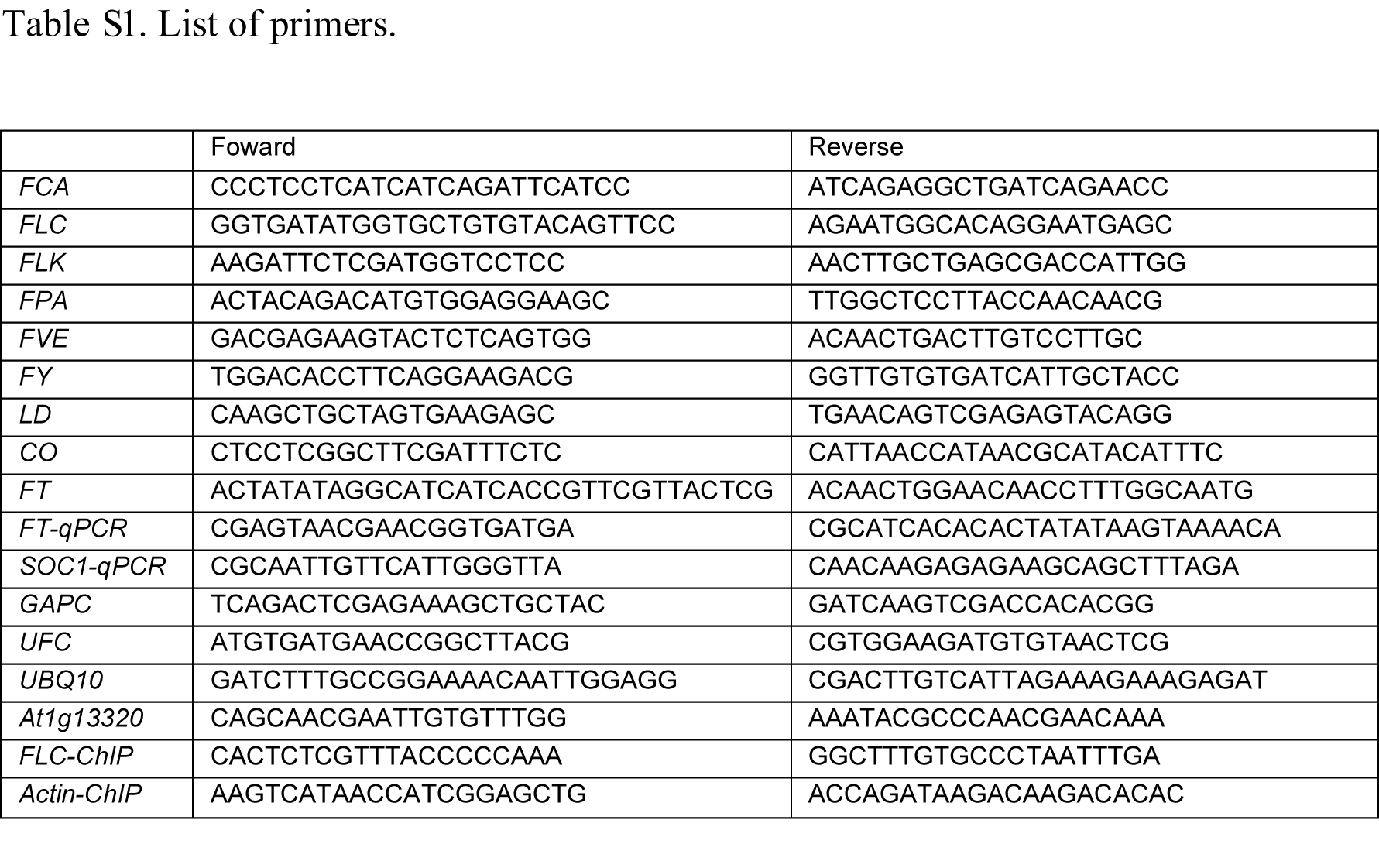

Supplement: Table S1 — List of primers. (TIF) [file pone.0017997.s003.tif]
